# Practical tips for navigating a program director transition

**DOI:** 10.12688/mep.19492.1

**Published:** 2023-01-10

**Authors:** Michael A. Gisondi, Laura Hopson, Linda Regan, Jeremy Branzetti

**Affiliations:** 1 Emergency Medicine, Stanford University School of Medicine, Stanford, CA, 94304, USA; 2 Emergency Medicine, University of Michigan School of Medicine, Ann Arbor, MI, USA; 3 Emergency Medicine, Johns Hopkins School of Medicine, Baltimore, MD, USA; 4 Emergency Medicine, Geisinger Health System, Danville, PA, USA

**Keywords:** Program Director; Residency; Fellowship; Transition; Search; Succession;

## Abstract

Residency and fellowship program directors profoundly impact trainees, institutions, and patient safety. Yet, there is concern for rapid attrition in the role. The average program director tenure is only 4-7 years, and that timeline is likely a result of burnout or opportunities for career advancement. Program director transitions must be carefully executed to ensure minimal disruption to the program. Transitions benefit from clear communication with trainees and other stakeholders, well-planned successions or searches for a replacement, and clearly delineated expectations and responsibilities of the outgoing program director. In this Practical Tips, four former residency program directors offer a roadmap for a successful program director transition, with specific recommendations to guide critical decisions and steps in the process. Themes emphasized include readiness for a transition, communication strategies, alignment of program mission and search efforts, and anticipatory support to ensure the success of the new director.

## Introduction

In 2022, 6,087 residency and fellowship programs participated in the U.S. National Residency Matching Program ( NRMP, 2022). By extension, there are at least that many program directors overseeing the training of the U.S. physician workforce. While program directors make up only a small percentage of the estimated 170,116 clinical faculty members across U.S. medical schools, their impact on trainees, institutions, and patient safety is profound ( AAMC, 2022; Kolarik *et al.*, 2018; Lam *et al.*, 2016). So critical is the role, the Accreditation Council for Graduate Medical Education (ACGME) requires programs to “demonstrate retention of the program director for a length of time adequate to maintain continuity of leadership and program stability” ( ACGME, 2022). Despite this mandate, there are concerns of rapid attrition for this important role and the resultant negative impact of attrition on resident training ( Brown & Gerkin, 2019; Fletcher *et al.*, 2020; O’Connor *et al.*, 2019; Stadler *et al.*, 2020).

Studies suggest that the average program director tenure is only 4–7 years ( Brown & Gerkin, 2019; Craig *et al.*, 2012; O’Connor *et al.*, 2019; Pallant *et al.*, 2019) and it is estimated that 11–14% of program directors leave their positions annually ( ACGME, 2018). While the reasons that program directors step down are multifactorial, research suggests that burnout or career advancement are the most likely determinants ( De Oliveira *et al.*, 2011). In one study, the high rate of turnover of internal medicine program directors was associated with voluminous administrative work, the view that the position is a stepping stone to other roles, and a lack of training for dealing with problem residents ( Beasley *et al.*, 2001). In addition, constant change management and complex accreditation standards negatively affect morale, add to overall workload, increase burnout, and cause attrition ( Choa *et al.*, 2022; Mullins & Gunderman, 2015; Yager *et al.*, 2022). Emotional exhaustion and depression have been observed in burned out program directors ( Psenka *et al.*, 2020). We believe these stressors affect program directors in most specialties and determine the context and projected timelines for them to step down.

Irrespective of cause, program director transitions occur in one of two ways. While some may happen abruptly and out of immediate necessity, most transitions can be carefully planned to ensure the least disruptive change for stakeholders and the program-at-large. Planned or not, we believe there are general principles that should be followed when a program director steps down. These reflect the need for thoughtful communication to staff and trainees, searches or successions that strategically align with program missions, and a shared understanding of the involvement of the outgoing program director. If a deliberate approach to this process is not followed, programs risk threats to stability, stakeholder cohesion, progress with ongoing initiatives, and quality of training.

In this Practical Tips, we provide program directors a roadmap and best practices for stepping down from the role. Our author team is composed of four former residency program directors with a combined 33 years of tenure, and our collective experiences of stepping down informed the recommendations that follow. We address program directors directly in this piece, although we believe that other education leaders will find the content valuable, as well.

## Tips

### Tip 1

**Figure 1.**
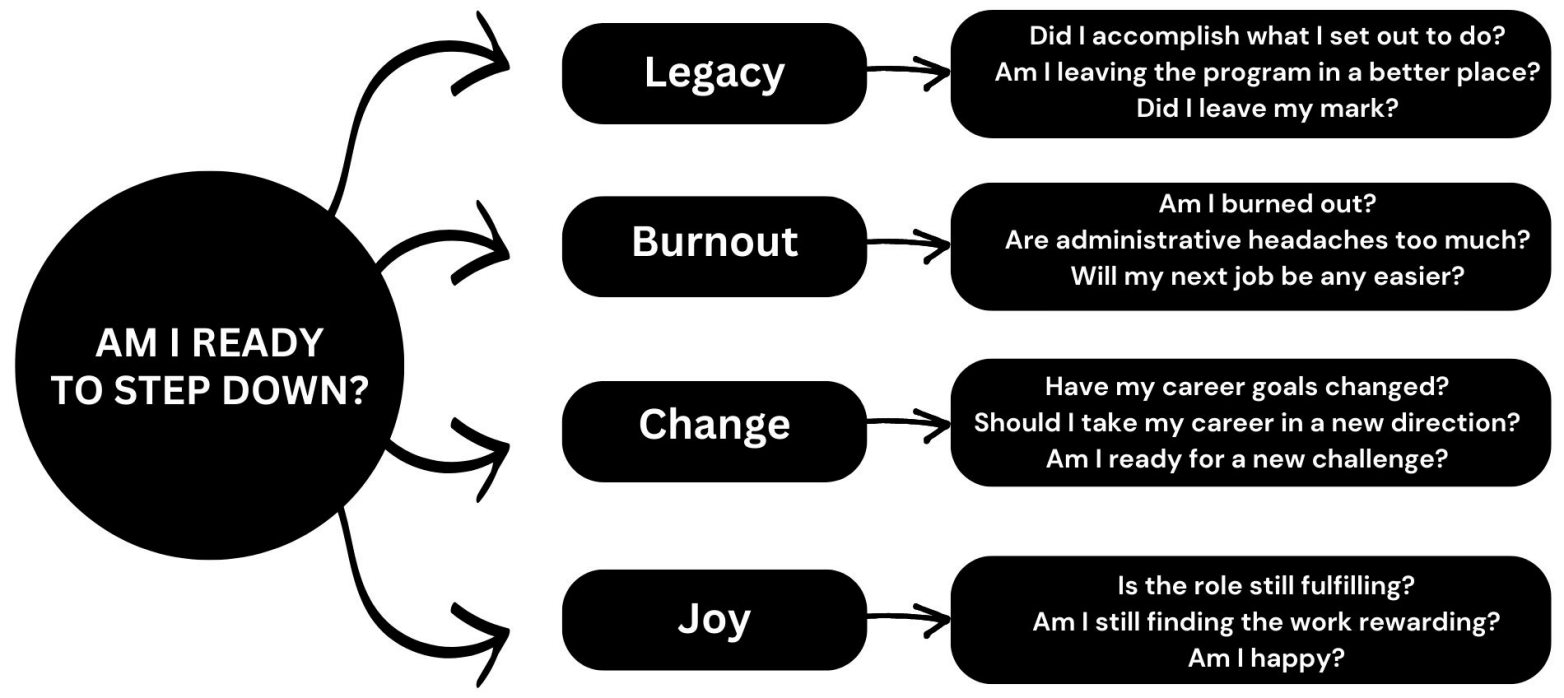
Am I ready to step down? Mind map of questions for uncertain program directors.

***Recognize when it is time to step down.*** There are many reasons that a program director may consider stepping down. Promotion to a new role is common, such as chair/vice chair or assistant/associate dean ( Papanagnou *et al.*, 2019). You must decide whether the new opportunities offered in those appointments are potentially more rewarding than your current role. If you are uncertain about remaining program director, reflect on the questions posed in Figure 1. In short, are you burned out? Is it time for a change? Have you left a legacy? Are you still finding joy in the role? ( De Oliveira *et al.*, 2011; O’Connor *et al.*, 2019; Schell, 2007; Yager *et al.*, 2022). Studies suggest that the retention of program directors is related to life-enriching work experiences such as counseling struggling residents, managing programs during traumatic circumstances, and overseeing residents from recruitment to board certification ( Yager *et al.*, 2022). However, it is common to enjoy working closely with residents while simultaneously feeling burnt out from administrative obligations ( Cullen *et al.*, 2021; De Golia *et al.*, 2022). Trading administrative headaches for professional goals may have been acceptable at the start of your tenure, but does it remain so now? This introspection is critical to recognizing when it is time to step down from the role.

### Tip 2

***Have a succession plan already in place.*** Program directors may choose to remain in their roles until replaced or they may step down immediately. In the latter case, proper succession planning will minimize disruptions ( Thompson & Augoustides, 2019). Always have a successor in mind, whether that be an assistant/associate director, a former program director still on faculty, or other leaders within your faculty. It is important to have these individuals identified well in advance of any unexpected/extended leaves or an abrupt transition ( Deitte *et al.*, 2021). Proactively include potential successors in program leadership efforts so they are versed in the management of the program should they need to quickly step into the role ( Dieter & Coombs, 2022). Such experiences will help them better understand the work of a program director and may even prompt interest in eventually assuming the role ( Mainiero, 2003). These experiences also allow opportunities for them to demonstrate their efficacy in the position and for an assessment of their candidacy. Likewise, it also allows you to identify who is *not* a suitable candidate.

### Tip 3

***Clarify expectations with your department chair.*** Clearly delineate expectations with your department chair during the transition, including timing of announcements and your roles and responsibilities. Given that many program directors continue in the position until a replacement is hired, it is essential to avoid being perceived as a ‘lame duck’ in your final months. Once the transition is announced, stakeholders may have less political appetite for you to enact significant changes to the program. Set expectations with your chair for support if you must make tough decisions about the program or trainees near the end of your tenure. Equally important is the need to negotiate the length of your remaining tenure with your chair ( Bessey *et al.*, 2002). You may not want to remain in the role indefinitely while a search process is executed, potentially stalls, or is unsuccessful ( Dieter & Coombs, 2022). That said, you may be the one asking to extend your tenure while you navigate unexpected delays in licensure, credentialing, academic appointments, or income at your new job. Lastly, clarify your level of involvement in the search for your replacement. Some chairs may prefer that you not participate at all, while others will want your guidance in identifying and interviewing potential candidates. The right level of involvement may be dictated by your chair, the circumstances of your transition, or your personal preference about assisting with the search.

### Tip 4

***Thoughtfully announce the transition to key stakeholders.*** Identify individuals who will be directly impacted by a change in program leadership and determine the best ways to notify them about the transition. Key stakeholders include the department chair and executive leadership team, the designated institutional officer, the residency leadership team, division chiefs and section directors, faculty members, residents and fellows, program alumni, the Graduate Medical Education (GME) Committee, the ACGME, and the human resources office ( Lypson & Simpson, 2011). The order in which you notify stakeholders is important. For instance, inform the department chair and designated institutional officer before other faculty members, and inform the chief residents before the other trainees. Tailor the announcement to each of these constituencies, allowing time for processing and questioning. Focus on the program’s continued commitment to excellence. It is *essential* that the trainees do not find out about your impending transition before you tell them personally. This announcement should be planned in collaboration with your department chair and done in person rather than by email. A follow-up message can then be sent to a larger audience, including faculty, clinical staff, and those trainees who may not have been present during the in-person announcement.

### Tip 5

***Be transparent with trainees and program applicants.*** Program director transitions are upsetting to current residents who value program stability, and they may believe that their program experience is tied specifically to you as their director ( Lam *et al.*, 2016). Change can be especially stressful for those who selected their program based on their positive interactions with you during recruitment; some may feel duped. Address their concerns openly in a town hall-style setting. Bring your department chair (and successor, if already chosen) to sit next to you during the discussion as a demonstration of stable department leadership. Describe the institutional commitment to the program’s future success and be honest about your reasons for stepping down. Transparent dialogue during the initial announcement can prevent gossip, blame, and anger later. Aim to over communicate, including individual check-ins with trainees if needed. If your successor is not known, describe the anticipated timeline for a replacement. Recognize that junior trainees will be worried that the philosophy and expectations of the program may change, while senior residents may be more concerned with practical issues such as who will provide their job references. Candidates to your training program will appreciate open and honest dialogue throughout their recruitment process, as well ( Rosmarin *et al.*, 2020). Expect candidates to ask difficult questions about the transition to all team members, so be sure everyone is prepared and messages align. Avoid announcing a planned transition immediately after a recruitment cycle, as incoming trainees will arrive angered about a perceived ‘bait and switch.’

### Tip 6

***Take care of the residency leadership team.*** Leadership transitions in academic medicine can be inherently stressful for faculty members, especially the direct reports of the outgoing leader. Expect the program director transition to cause anxiety for assistant/associate directors and administrative team members. Directly acknowledge the uncertainty and support the team through the transition ( Grady, 2021; Rowland *et al.*, 2022). Review the transition plan with the team as soon as possible, including projected timeline, potential reassignment of roles, information about the upcoming search, and communications strategies. Reinforce their value by soliciting their insights about the position and the needs of the program. Consider that one or more of your team members may anticipate becoming the next program director; it is imperative to inform assistant/associate directors or other potential successors if they will not be seriously considered for the role. Others may use the transition to reappraise their own interest in continuing as an associate director. Transparent and direct communication fairly addresses such expectations and reactions. 

### Tip 7

***Revisit the program mission.*** Leadership transitions are a natural time to reflect on the program’s mission, vision, and values ( Shappell *et al.*, 2018). Such exercises help stakeholders consider the future instead of perseverating upon the past. Stakeholder engagement is critically important for their insights and their buy-in for change; collaborative involvement reinforces their sense of ownership and investment in the program. This can be accomplished through retreats, town halls, formal program evaluations, or external reviews. Use the mission statement to guide an honest assessment of the program and a shared vision for the future. Why does the program exist? What program aims distinguish it from other training programs? ( Bhat-Schelbert *et al.*, 2004) What are its strengths and potential areas for growth under new leadership? Is it resourced and supported appropriately to meet its current goals? Search processes will be more successful if candidates espouse your stated mission and values; however, that requires that you can clearly articulate such defining characteristics.

### Tip 8

***Describe the desired characteristics of a new program director.*** Before a search process can begin, solicit the desired characteristics of a new director from stakeholders -- personality traits, leadership styles, etc. ( Borsa, 2005). For instance, if you used a gentle and warm leadership approach with your trainees, a successor with a more firm or intentionally distant style may be culturally disruptive to an otherwise stable program. However, excessive concerns about programmatic change will make it challenging to imagine how a new and different leader could be equally successful. A search for your ‘clone’ may result in a narrow and biased process ( White-Lewis, 2020). A formal program evaluation, such as a SWOT analysis, can identify the non-negotiable traits, skills, experiences, and differences that prospective candidates must possess. No candidate will check all the boxes, however. Consider building systems and teams around a new hire who may have weaknesses in some areas and clear strengths in others.

### Tip 9

***Execute a strategic and inclusive search.*** Program directors advancing to roles such as vice chair may be expected to conduct a search for their replacement. Effective searches are time-consuming and effortful. The key principles are to clearly describe the role, maximize the reach of your search, and ensure your candidate evaluation process is fair and unbiased ( Pitts, 2017; Turner, 2002). Predefined candidate merits will provide the recruitment team with clarity about the successor they seek, and such descriptions have been shown to mitigate bias in the process ( Uhlmann & Cohen, 2005). Begin by crafting the job advertisement to reflect the desired technical skills and characteristics of a new director, going beyond standard boilerplate language. The advertisement should reflect the program mission and describe any high-priority initiatives that are ongoing. Post ads broadly in professional society publications, especially those that serve physicians underrepresented in medicine or women ( Fraser & Hunt, 2007). Leverage social media and encourage peers to post the call for applicants. To ensure a diverse pool of candidates, assemble a search committee with diverse perspectives and backgrounds and have them engage in direct outreach to qualified contacts. This committee should complete implicit bias training, and interview questions and candidate evaluations should be screened for content that could be gendered or biased ( Cahn, 2017). A snowball technique of outreach to potential candidates can be useful when offers to interview are declined ( Fraser & Hunt, 2007). Internal candidates should be evaluated using the same rubrics designed for external candidates. Search information should remain confidential until candidates discuss the opportunity with their current employers; only then should you check references. Notify non-selected candidates early.

### Tip 10

***Respond to a failed search.*** Even well-planned national searches sometimes fail. If your offer isn’t accepted, listen carefully to the needs of the candidate and be prepared to negotiate. Desired candidates may be reluctant to move for family reasons and qualified individuals will likely have multiple job offers. Candidates may withdraw from the process at the last minute, adding to the emotional stress of the transition ( Cohen, 2004). Review your rank list of additional candidates and your best alternatives to a failed search. If your initial pool of candidates falls through, then a period of reflection and a return to the mission and values of the program are warranted. You might conduct a repeat search for both external and internal candidates or may simply require a reassessment of the salary and benefits offered in the initial search. Whatever the response to a failed search, you must know whether the outcome will delay your transition. Who will direct the program during this time? Do you remain in your role? For how long? Do prior arrangements with your chair require renegotiation? Consider requesting a retention package in agreement for remaining in the role for an extended time and be ready to name an interim director in consultation with your department chair if the situation demands it. Provide honest updates to stakeholders in the event of a failed initial search and offer a clear plan forward.

### Tip 11

***Anticipate problems during the transition.*** Good planning can limit the number of problems encountered during the transition, yet expect the unexpected. Internal successors should offload previous assignments to dedicate adequate time to their new role, yet they may be resistant to give up meaningful work. Consider assuming some of their previous administrative duties temporarily to help them manage their time. Internal candidates who were passed over for your position may be angry, embarrassed, or emotionally detached. Your successor will need support when building their new leadership team, especially if they want to transition assistant/associate program directors out of their roles ( Turner, 2002). Some team members may abruptly step down if they didn’t support the chosen candidate. Your best strategy is to remain positive about the program while thoughtfully helping your former team members as they seek new opportunities. External candidates can be met with animosity if stakeholders wanted an internal candidate to be chosen. There are inherent risks of taking an outside candidate for the role who doesn’t understand or appreciate the existing program culture; even when they are prepared, they may not be able to affect necessary changes until stakeholders believe that change is needed or begin to trust them. Additionally, programs that are in crisis – such as those with unanticipated leadership changes or an accreditation threat – represent complicated challenges for the chair and the institution. Interventions by GME leadership or external consultants are warranted.

### Tip 12

***Survive the process and let go.*** Transitioning from the program director role can be challenging and stressful, especially when you may still find it enjoyable and rewarding ( Schell, 2007). It can drain time and energy, and test emotions. You may feel that your connection to the trainees is at risk and that your successor can’t care for them as you did. You might have unfinished goals for the program and regret your decision to step down. You may find yourself in a ‘meaning vacuum’ that accompanies massive life disruptions, making you feel frightened, overwhelmed, and stuck ( Feiler, 2020). Let go. Focus on your new professional goals in a manner that does not include your program director identity. What’s next for you? Consider retaining a professional coach to guide you in your new pursuits and rely on mentors and family as you embark on your next journey. Give your replacement the freedom to leverage their new political capital in the role, determine the composition of their team, and establish their own relationships with the trainees. If you remain a faculty member in your department, step aside and let your successor direct the program. It may take them years to establish themselves in the role and demonstrate measurable outcomes, so be patient. Remain a trusted advisor if that is their request, otherwise be silent and offer support only when asked. Allow failures with a reasonable safety net. Finally, regard the time, effort, and outcomes of your program director tenure as a foundation built for your successor – and your legacy as one to be proud of. To new beginnings!

## Conclusion

Program directors have an expected tenure, and their transitions from the role should be strategically managed to ensure a smooth change in leadership. Communications with all stakeholders must be transparent, timely, and thoughtfully planned. Formal searches for a replacement director allow for reflection on the program mission and its values. Outgoing program directors should be available for assistance but should otherwise be unobtrusive to the efforts of their replacements.

## Data Availability

No data are associated with this article. Michael A. Gisondi, MD (Stanford School of Medicine), Jeremy Branzetti, MD, MHPE (Geisinger Health System). Linda Regan, MD, M.Ed. (Johns Hopkins University School of Medicine), and Laura R. Hopson, MD (University of Michigan Medical School) are previous residency directors in emergency medicine with a combined tenure of 33 years.
