# Supplementary material for: Practical tips for navigating a program director transition
Source: MedEdPublish (2016). 2023 Jan 10;13:3. [Version 1] doi: 10.12688/mep.19492.1 (PMC9989543; doi:10.12688/mep.19492.1)
Supplement: Peer review history

# Peer review: Reviewer response for version 1

**DOI:** 10.21956/mep.20885.r32869

**Published:** 2023-03-06

**Authors:** Stephen J. Wolf

**Affiliations:** 1 Department of Emergency Medicine, Denver Health, University of Colorado School of Medicine, Denver, CO, USA

Thank you for the opportunity to review MedEDPublish19491 manuscript for MedEdPublish. Having served as a program director, Director of Education, and now departmental Director of Service, I fully understand the critical importance of this topic of career transition for Program Directors and Departments

**Overall**:

 I liked the practical tip approach and thought the manuscript was well written. I find the content and advice accurate, appropriate and meaningful for both program directors and departments. I have a few edits or comments that if addressed, I think would make the article more appropriate and valuable to its intended audience.

- Intro & others: I would be sure that wherever applicable the tips are directed not only towards the program director but also the department/departmental leadership who are also navigating the transition.
  Eg., pg 3 first line of para 4 change to  “…we provide program directors and departmental leadership a roadmap…” I would carefully review for other instances.
- Tip 1: I would add a concept of recognizing when you may no longer be bringing value to the program in a productive and constructive way. Sort of ‘knowing when you are stale to the position.’
  I wonder if this concept could even be included in figure 1 – ‘Value -> am I still authentically bringing value and meaning to the program?’
- Tip 5: I would consider included not just being transparent but providing help and support where needed in the bolded portion.
- Conclusion: I would more specifically call out the residents.
  Eg., ‘…. Their transitions from the role should be strategically managed to ensure a smooth change in leadership, continued success of the residency program, and ongoing wellness of the residents’ (or something like that…)

Thank you for the opportunity to participate in this review.

If evidence from practice is presented, are all the underlying source data available to ensure full reproducibility?

Not applicable

Is the topic of the practical tips discussed accurately in the context of the current literature

Yes

Are all factual statements correct and adequately supported by citations?

Yes

Are the conclusions drawn balanced and justified on the basis of the presented arguments?

Yes

Are arguments sufficiently supported by evidence from the published literature and/or the authors’ practice?

Yes

Reviewer Expertise:

Leadership, medical education and clinical policies

I confirm that I have read this submission and believe that I have an appropriate level of expertise to confirm that it is of an acceptable scientific standard.

# Peer review: Reviewer response for version 1

**DOI:** 10.21956/mep.20885.r32861

**Published:** 2023-02-16

**Authors:** Erica Shaver

**Affiliations:** 1 Department of Emergency Medicine, West Virginia University School of Medicine, Morgantown, WV, USA

Overall, I perceive this article as very well done and reflective of the current literature regarding the program director role, responsibilities, as well as factors related to burnout and turnover, hinging on the experiences of 4 well known authors and role models in emergency medicine--which supports the credibility of the contents. It does a nice job suggesting why there is a need to navigate a PD change to begin with, with most common reasons that PD's leave their PD roles due to 1. exhaustion and burnout in the current position or 2. being promoted up and out of the role. It then goes on to fairly clearly outline every step in the process, with each step seemingly generalizable enough that they could be applicable to PD's transitioning roles, regardless of institution. 

 One small thing I was thinking as I read through the points was that point #2, have a succession plan in place, suggests that every PD should have a "go to" person to start fulfilling the role if and when they want to step down--as if this person will be given the PD role when it's time for the transition without any additional questions being asked--sort of an "assumed position". Then when we move to tip 9, we talk about conducting an extensive search looking at both internal and external candidates for the role, treating both equally, and really looking for the person that fits the role the best. So my thought would be how do these points correlate? If you suggest that there should be an automatic PD successor ready, would this person be initially just referred to as *interim* PD while the more formal search is being conducted? Or is it supposed to be understood that that is the suggestion here, even without it being fully spelled out? Perhaps just clarifying the role of the PD successor appointed well in advance of the PD leaving the role, and how they serve in the role until a potentially new person is appointed may clear that point up a bit for the readers. To me, if you appoint a PD successor, you are choosing someone internally who will be mentored to take the role. If then an extensive search is conducted, this sort of defeats the purpose of appointing a successor ahead of time and training them up, outside of them being strictly placed as a PD interim in the case of sudden PD loss. 

 Otherwise, no major suggestions, and I really enjoyed reading the article!

If evidence from practice is presented, are all the underlying source data available to ensure full reproducibility?

Yes

Is the topic of the practical tips discussed accurately in the context of the current literature

Yes

Are all factual statements correct and adequately supported by citations?

Yes

Are the conclusions drawn balanced and justified on the basis of the presented arguments?

Yes

Are arguments sufficiently supported by evidence from the published literature and/or the authors’ practice?

Yes

Reviewer Expertise:

medical education, pass/fail step 1, rural emergency medicine, lower 1/3 SLOE

I confirm that I have read this submission and believe that I have an appropriate level of expertise to confirm that it is of an acceptable scientific standard.
